# Analysis of the intestinal microbiota using SOLiD 16S rRNA gene sequencing and SOLiD shotgun sequencing

**DOI:** 10.1186/1471-2164-14-S5-S16

**Published:** 2013-10-16

**Authors:** Suparna Mitra, Karin Förster-Fromme, Antje Damms-Machado, Tim Scheurenbrand, Saskia Biskup, Daniel H Huson, Stephan C Bischoff

**Affiliations:** 1 Singapore Centre on Environmental Life Sciences Engineering, Nanyang Technological University, Singapore 637551; 2 Department of Nutritional Medicine, University of Hohenheim, Stuttgart, Germany; 3 Center for Bioinformatics, University of Tübingen, Tübingen, Germany; 4 CeGaT GmbH, Tübingen, Germany

**Keywords:** Metagenomics, Intestinal Microbiota, Next-Generation Sequencing, SOLiD Mate-Pair Sequencing, Human Fecal Sample

## Abstract

**Background:**

Metagenomics seeks to understand microbial communities and assemblages by DNA sequencing. Technological advances in next generation sequencing technologies are fuelling a rapid growth in the number and scope of projects aiming to analyze complex microbial environments such as marine, soil or the gut. Recent improvements in longer read lengths and paired-sequencing allow better resolution in profiling microbial communities. While both 454 sequencing and Illumina sequencing have been used in numerous metagenomic studies, SOLiD sequencing is not commonly used in this area, as it is believed to be more suitable in the context of reference-guided projects.

**Results:**

To investigate the performance of SOLiD sequencing in a metagenomic context, we compared taxonomic profiles of SOLiD mate-pair sequencing reads with Sanger paired reads and 454 single reads. All sequences were obtained from the bacterial 16S rRNA gene, which was amplified from microbial DNA extracted from a human fecal sample. Additionally, from the same fecal sample, complete genomic microbial DNA was extracted and shotgun sequenced using SOLiD sequencing to study the composition of the intestinal microbiota and the existing microbial metabolism. We found that the microbiota composition of 16S rRNA gene sequences obtained using Sanger, 454 and SOLiD sequencing provide results comparable to the result based on shotgun sequencing. Moreover, with SOLiD sequences we obtained more resolution down to the species level. In addition, the shotgun data allowed us to determine a functional profile using the databases SEED and KEGG.

**Conclusions:**

This study shows that SOLiD mate-pair sequencing is a viable and cost-efficient option for analyzing a complex microbiome. To the best of our knowledge, this is the first time that SOLiD sequencing has been used in a human sample.

## Background

In recent years, next generation sequencing has revolutionized the field of metagenomic analysis. While metagenomic analysis using the gold standard of Sanger sequencing stands for time-consuming cloning of the DNA of interest and high costs per base, this technique has the advantage of long read lengths of very high quality. Three main advantages of next generation technologies are a much lower cost per base, the omission of a cloning step, and a huge amount of sequencing data per run. Currently, four different next generation sequencing systems dominate the market: the Roche 454 Genome Sequencer FLX System, the Illumina HiSeq, the Ion Torrent, and the Applied Biosystems SOLiD sequencer. We will refer to these three technologies as 454, Illumina and SOLiD sequencing, respectively. The 454 system was the first commercially available next generation sequencing platform and was introduced in 2004 [1]. The Illumina genome analyzer followed in 2006 [1] and the SOLiD sequencer in 2007 [1]. Of these three systems, the SOLiD system produces the highest amount of data per run at the lowest costs per base.

The primary field of application of next generation sequencing technologies is structural variant discovery by resequencing of targeted regions of interest or whole genomes, de novo sequencing of whole genomes of microorganisms, the analysis of transcriptomes, and the analysis of metagenomes. A number of metagenomic projects have been performed using the 454 sequencer, studying such environments as deep-sea water [2], glacier ice [3], glacier foreland [4], forest soil [5], and the human gut [6]. Increasingly, Illumina sequencing is becoming the work-horse of metagenomic sequencing [7,8]. To date, no metagenomic studies recruiting human patients have been published based on SOLiD sequencing. A comparison of the three next generation sequencing systems based on analyzing the cod transcriptome is reported in [9]. The fact that SOLiD sequencing has not yet been used often in metagenomics can be attributed to the short read lengths produced by the technology, and also to the fact that the SOLiD sequencer produces reads in "color space" that must be translated into DNA reads. The latter problem is error prone in the absence of an appropriate reference sequence. In the present study we used the Applied Biosystems SOLiD sequencer for sequencing the metagenome of a human fecal sample by 16S rRNA sequencing and also by shotgun sequencing of mate-pair libraries to investigate the suitability of SOLiD sequencing for metagenome sequencing. Furthermore, we performed 16S rRNA gene sequencing by traditional Sanger sequencing and 454 pyrosequencing of the V2 region of the 16S rRNA gene to verify this new application of SOLiD sequencing. The analysis of the sequencing data was performed using primarily the MEGAN 4 program [10]. To the best of our knowledge, this is for the first time that a metagenome analysis using human samples was performed using next generation SOLiD sequencing.

## Methods

### Sample collection and isolation of microbial DNA

The stool sample was collected in a stool collection tube with DNA stabilizer (Invitek, Berlin, Germany), returned to the analysis lab within one day, and stored at -80 °C until use. Microbial DNA was isolated using the Invitek PSP^® ^Spin Stool DNA Plus Kit (with lysis enhancer) as described by the manufacturer. The single stool sample used in this study derived from a 65 years old obese female (BMI 47.9 kg/m^2^). The study protocol was part of a multicenter clinical trial, research project "Obesity and the gastrointestinal tract" (ClinicalTrials.gov identifier: NCT01344525), approved by the ethics committee of the University Hospital of Tübingen, Germany. Informed consent was obtained prior to participation.

### Amplification and cloning of 16S rRNA genes for Sanger sequencing

Multiple replicate PCR reactions were performed from the fecal DNA sample. Each 50 μL reaction contained 100 ng template DNA, 0.3 μM of the forward universal bacterial primer 8F (5'-AGAGTTTGATCCTGGCTCAG-3'; *Escherichia coli *positions 8 to 27) [11], 0.3 μM of the reverse universal primer 1391R (5'-GACGGGCGGTGWGTRCA-3', *Escherichia coli *positions 1391 to 1407) [12], 1 U Platinum *pfx *DNA Polymerase (Invitrogen), 1 × *pfx *amplification buffer, and 0.3 mM of each dNTP. Cycling conditions were 94 °C for 2 min, followed by 30 cycles of 94 °C for 15 sec, 55 °C for 30 sec, and 68 °C for 120 sec, following a final elongation step at 68 °C for 10 minutes. Replicate PCRs were pooled and purified (Roche High Pure PCR product purification kit). Full-length 16S rRNA gene amplicons (1.3 kb) were cloned into pCR-Blunt II-TOPO (Zero Blunt^® ^TOPO^® ^PCR Cloning Kit, Invitrogen), and the ligated DNA was transformed into *E. coli *TOP10 (Invitrogen). 960 clones were selected and plasmids for sequencing were amplified using the Illustra TempliPhi Amplification Kit (GE Lifesciences). Plasmid inserts were sequenced by Sanger sequencing bi-directionally employing vector-specific primers (M13 forward and reverse) using an ABI BigDyeTerminator and 3730xl capillary electrophoresis.

### Amplification of the 16S rRNA-V2-region for pyrosequencing

Multiple replicate PCR reactions were performed from the fecal DNA sample. Each 50 µl reaction contained 100 ng template DNA, 0.3 µM of a modified universal bacterial primer 8F [11] [5'-CGTATCGCCTCCCTCGCGCCATCAG**ACGCTCGACA***AGAGTTTGATCCTGGC TCAG*-3'; composite of 454 primer A (underlined), key nucleotides (TCAG), a unique 10 bases barcode (bold) and the universal bacterial primer 8F (italics)], 0.3 µM of a modified universal bacterial primer 338R [13] [5'-CTATGCGCCTTGCCAGCCCGCTCAG*GCTGCC TCCCGTAGGAGT*-3'; composite of 454 primer B (underlined), key nucleotides (TCAG), and the universal bacterial primer 338R (italics)], 1.25 U Prime STAR HS DNA Polymerase (Takara), 1 × PrimeSTAR buffer, and 0.2 mM of each dNTP. Cycling conditions were 95 °C for 2 min, followed by 30 cycles of 95 °C for 30 sec, 54 °C for 20 sec, and 72 °C for 60 sec, following a final elongation step at 72 °C for 10 minutes. Replicate PCRs were pooled and purified (Roche Agarose Gel DNA Extraction Kit). Pyrosequencing was carried out on a GS Junior system following the manufacturer's recommendations (454 Life Science, Roche) and was performed by Eurofins MWG, Ebersberg, Germany.

### Amplification of 16S rRNA genes for SOLiD sequencing

Multiple replicate PCR reactions were performed from the fecal DNA sample. Each 50 μL reaction contained 100 ng template DNA, 0.2 μM of the forward universal bacterial primer 8F (5'-AGAGTTTGATCCTGGCTCAG-3') [11], 0.2 μM of the reverse universal primer 1391R (5'-GACGGGCGGTGWGTRCA-3') [12], 1.25 U PrimeSTAR HS DNA Polymerase (Takara), 1 × PrimeSTAR buffer, and 0.2 mM of each dNTP. Cycling conditions were 95 °C for 2 min, followed by 30 cycles of 95 °C for 30 sec, 55 °C for 20 sec, and 72 °C for 150 sec, following a final elongation step at 72 °C for 10 minutes. Replicate PCRs were pooled and purified as described above.

### Library preparation for SOLiD Long-Mate-Paired sequencing

For SOLiD Long-Mate-Paired 16S rRNA gene and microbial DNA shotgun sequencing, (a) 1000 ng of amplified 16S rRNA gene and (b) 5000 ng of DNA isolated from feces was used, respectively. Long-Mate-Paired libraries with Mate-Paired distances of 300 - 900 bp apart from (a) the 16S rRNA gene sample and (b) the whole microbial DNA were generated by randomly shearing in a microTube format using the Covaris™ S2 sonicator, according to the Applied Biosystems mate-paired library construction protocol (version 1, March 2010) with minor modifications. For fragmentation the following protocol was used: Duty Cycle 5%, Intensity: 3, Cycles per Burst: 200 at 4 °C. Fragmentation times were adjusted to (a) 60 sec and (b) 15 sec to create libraries with Mate-Paired reads 300-900 bp apart (with a maximum at 800 bp). To achieve completion of the microbiome, no size-selection was performed. Finished libraries were clonally amplified on paramagnetic beads using 10 cycles, deposited onto a glass slide, and sequenced according to standard Applied Biosystems protocols for the SOLiD System, using the SOLiD™ 4 System.

Sequencing has been done at three stages: Sanger sequencing using 16S samples, SOLiD sequencing using both 16S and shotgun samples. We will refer to these datasets as 'Sanger' dataset, '16S-SOLiD' dataset and 'Shotgun-SOLiD' dataset.

### Data Processing

SOLiD sequencing produces reads in 'csfasta' format and in two files consisting forward and reverse reads. Thus the first step of data processing involved conversion of reads to 'fasta' format. Using knowledge of the first base of each SOLiD read, we translated reads from color space to DNA based on the standard SOLiD color space translation table. In this approach, an error in the color sequence will scramble the remaining sequence, so we discarded all reads that contained any base with a quality value less than 18, for both '16S-SOLiD' dataset and 'Shotgun-SOLiD' dataset. Color space reads were translated into DNA using the assumption that the first sequenced base is a T in the forward mates and G in the reverse mates [14].

All resulting 16S sequences were aligned against the SILVA ribosomal RNA sequence database [15] using the program BLASTN [16], default settings. BLAST was run using default settings so as to initially obtain as many matches as possible, even ones that have quite low statistical significance. More stringent parameters were used in the downstream analysis to focus on significant matches.

We considered all the reads of length 40 bp or above from both forward and reverse files, together with their mates. We removed the adapters from both the files ('T' from the forward mates and 'G' from the reverse mates) and aligned all of these sequences against NCBI-NR database of non-redundant protein sequences [17] using BLASTX, default settings.

Sanger and 454 sequencing produces reads in 'fasta' format, and these sequences are directly used without any pre-processing. All 16S sequences obtained using Sanger and 454 technology were aligned against the SILVA ribosomal RNA sequence database [15] using the program BLASTN [16], default settings.

After performing the BLAST comparison, both output files of Sanger and SOLiD data were imported and analyzed using the paired-end protocol of MEGAN [10], while the 454 reads was processed using the single-read protocol of the program. When processing the BLAST files by MEGAN we used parameter settings of *Min Score = 90*, *Top Percent = 10 *and *Min Support = 5*, for 16S sequences and *Min Score = 35*, *Top Percent = 10 *and *Min Support = 25 *for shotgun sequences. The reason that we used more stringent parameters for the analysis of 16S sequences is that the SILVA database covers a wider range of sequence diversity [18], is smaller and better curated than NCBI-NR, and thus it is easier to obtain high-scored alignments. When importing BLASTN output files produced by comparing against the SILVA database some adjustments need to be made (as described in [19]) in comparison to the default case of regular BLASTX files compared against the NCBI-NR database. After importing datasets in MEGAN we obtain tree-view of each data mapped onto NCBI taxonomy based on our selected threshold. Some reads which do not have any match to the respective database are placed under "No hit" node, and some reads that are originally assigned to a taxon that did not meet our selected threshold criterion are placed under "Not assigned" category.

Finally all the files were compared based on their taxonomic content.

Furthermore we performed a functional analysis of the SOLiD shotgun sequences using the SEED classification [20], based on the given BLASTX output file. In such an analysis reads are assigned to functional roles in the SEED classification. To obtain a tentative pathway analysis, we performed an analysis based on KEGG (Kyoto Encyclopedia for Genes and Genomes, [21]). In more detail, reads were mapped onto KEGG orthologous groups using MEGAN.

### Data deposition

The sequence data obtained in this study have been deposited in the DNA Data Bank of Japan under the BioProject accession number PRJDB86.

## Results

### Sanger sequencing

Sanger sequencing resulted in 1242 reads of 16S rRNA gene sequences ('Sanger'-dataset). After aligning the reads against SILVA database, using BLASTN, we imported the results into MEGAN, where 1228 reads could be assigned. Surprisingly, we found a high abundance of *Cyanobacteria *in the Sanger data set.

### 454 sequencing

454 sequencing resulted in 72,571 reads of 16S rRNA gene sequences ('16S-454'-dataset). After aligning the reads against the SILVA database, using BLASTN, we imported the results into MEGAN, where 72,350 reads could be assigned. The abundance of *Cyanobacteria *was much lower in 454 sequences compared to the Sanger sequences. Furthermore, we detected slightly more *Bacteroidetes *than *Firmicutes *in this dataset, and also phyla being less abundant compared to *Bacteroidetes *and *Firmicutes *such as *Verrucomicrobia *and *Actinobacteria *easily overlooked when using Sanger sequencing. *Proteobacteria *and *Clostridiaceae *were only detectable at a low level by this approach.

### SOLiD sequencing

**16S sample: **After filtering low quality sequences (during conversion from 'csfasta' to 'fasta', as mentioned above) we obtained 3,767,260 reads (2,155,456 forward and 1,611,804 reverse) for 16S samples ('16S-SOLiD' dataset). All sequences were blasted against the SILVA database and then imported into MEGAN, leading to assignments for 2,530,912 reads.

**Shotgun sample: **The above-mentioned conversion from 'csfasta' to 'fasta' format with quality filtering resulted in 10,764,512 forward and 9,997,372 reverse-reads for the 'Shotgun-SOLiD' dataset. Of these 3,168,307 forward and 4,577,127 reverse reads have length 40 bp or above. There were 791,321 mate pairs in which both reads had length of 40 bp or more. Further, there were 861,344 mate pairs in which only the forward read has length 40 bp or more and 1,798,245 matepairs in which only the reverse read had a length of 40 bp or more. In total, we considered 3,450,910 mate sequences or a total of 6,901,820 sequences for which at least one of the mates was at least 40bp long (for details see Table 1).

**Table 1:** Details of sequence reads of 'Shotgun-SOLiD' dataset.

Data type (shotgun sample)	File consisting forward reads	File consisting reverse reads
**Fasta file after quality filter**	10,764,512	9,997,372

**Reads of length 40+ bp **	3,168,307	4,577,127

**Reads where both the mates are 40+bp**	791,321	791,321

**Mates where one read is 40+bp other is <40bp**	861,344 forward (40+bp) reads has <40bp reverse mates	1,798,245 reverse (40+bp) reads has <40bp forward mates

**Total number of reads processed for BLAST**	3,450,910	3,450,910

After adapter removal, all of these sequences were aligned against the NCBI-NR database using BLASTX and imported into MEGAN. Using the above-mentioned thresholds 1,100,372 reads could be assigned to some node in the NCBI taxonomy.

**Figure 1:**
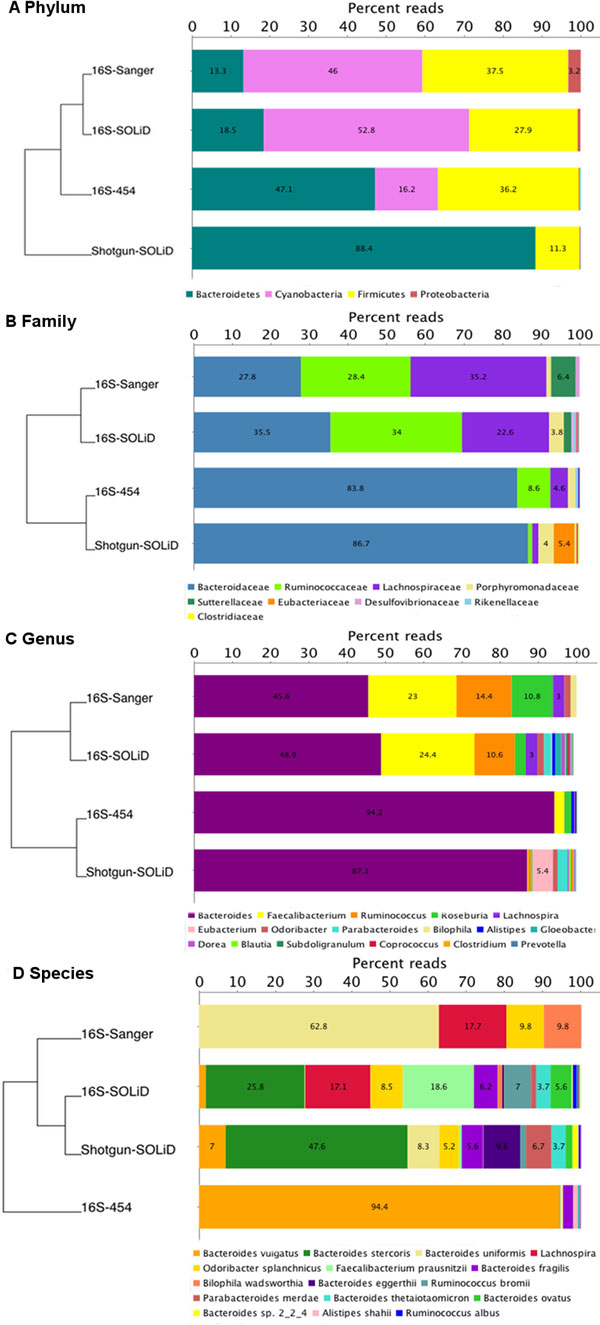
**Comparison of abundances of bacterial groups on different taxonomic levels obtained by 'Sanger', '16S-454', '16S-SOLiD' and 'Shotgun-SOLiD' sequencing**. (A) Phylum level, (B) class level, (C) genus level, and (D) species level. Columns are organized according to clustering results based on normalized Euclidean distance analysis of the phylogenetic tree on each taxonomic level, as displayed on the left.

A comparison of main abundances of bacterial groups on four taxonomic levels derived from the different sequencing technologies is shown in Figure 1. Additional file 1 shows the tree view of normalized comparison of the data obtained from these four methods. We have highlighted the nodes (showing sum and assigned read numbers) that are used to create Figure 1. Further when judged, as overview in Figure 1, 16S-Sanger and 16S-SOLiD generally look similar to each other except 'species' level, this is because using 16S-SOLiD we have much more reads compared to Sanger, and that helped us to achieve more species richness.

### Comparison of 16S and shotgun samples obtained using SOLiD technology

**Figure 2:**
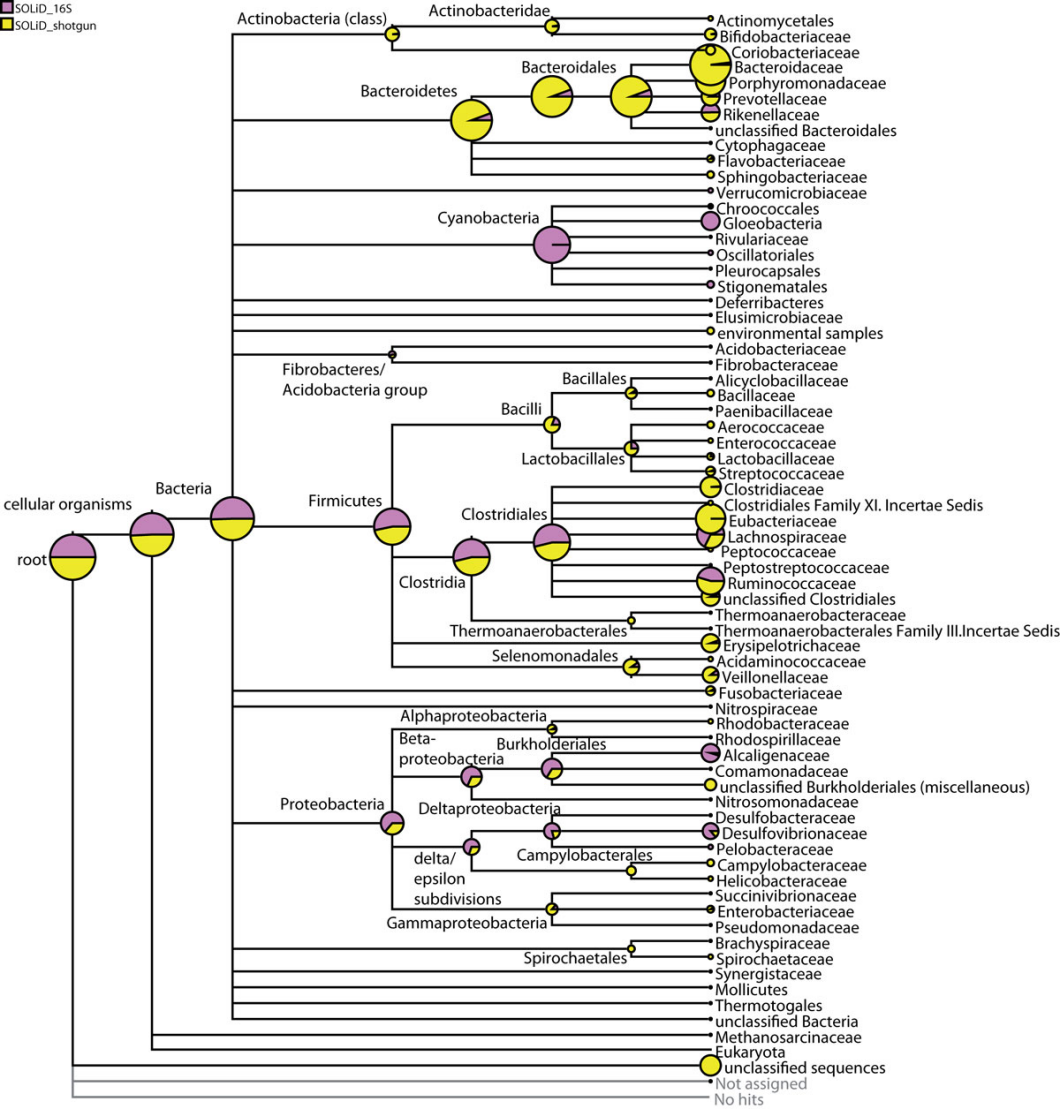
**Normalized comparison result obtained using MEGAN for '16S-SOLiD' dataset and 'Shotgun-SOLiD' dataset**. Normalized comparison result obtained using MEGAN for '16S-SOLiD' dataset (magenta) and 'Shotgun-SOLiD' dataset (yellow). '16S-SOLiD' dataset is blasted against the SILVA database and 'Shotgun-SOLiD' dataset is blasted against the NCBI_NR database. The tree is collapsed at 'family' level of NCBI taxonomy. Circles are scaled logarithmically to indicate the number of assigned of reads.

Figure 2 shows a normalized comparative tree-view of the assignments at 'family' level of NCBI taxonomy. Beside information about the composition of the microbiome (as is the case with 16S rRNA sequences), the shotgun DNA includes information about the encoded proteins. While a higher percentage of the 16S rRNA sequences could be taxonomically assigned, the composition of the microbiota inferred by both approaches was comparable. However, there were microbial species that outweighed in one approach compared to the other. In shotgun sequencing, more *Actinobacteria*, *Bacteroidetes*, *Bacillales, Lactobacillales*, *Clostridiaceae*, *Eubacteriaceae*, *Gammaproteobacteria*, *Selenomonadales *and *Fusobacteriacae *were detectable. On the other hand, in 16S rRNA gene sequencing, we found confirmation for the high abundance of *Cyanobacteria*. In contrast, we could find only a few reads assigned to *Cyanobacteria *in shotgun sequencing. On the one hand, this over-representation could be caused by preferential amplification of the 16S rRNA genes of *Cyanobacteria *as argued in the Sanger sequencing results section. Furthermore, we found more reads that map to *Verrucomicrobiacea*, *Clostridiales *and *Proteobacteria *in 16S rRNA gene sequencing than in shotgun sequencing. The two major phyla in the intestinal microbiome, the *Firmicutes *and *Bacteroidetes*, are represented differently by the two approaches. While 16S rRNA sequencing revealed more *Firmicutes*, shotgun sequencing resulted in more *Bacteroidetes*. This difference could be due to artifacts of the amplification of 16S rRNA genes.

The results reported here are based on using all mate pairs for which at least one of the two reads has a length of 40 bp or more. If one would consider only those mate pairs, for which both reads have a length of at least 40 bp, then the number of reads considered would drop by 75%, resulting in a huge decline of computational requirements, but one will lose 33% of assigned reads (see Additional file 2) which leads to 21 more species. Hence, in some studies it may be sufficient to only consider mate pairs in which both reads are longer than 40 bp, if there are plenty of such reads.

### Comparison of 16S samples from three technologies (Sanger, 454 and SOLiD)

**Figure 3:**
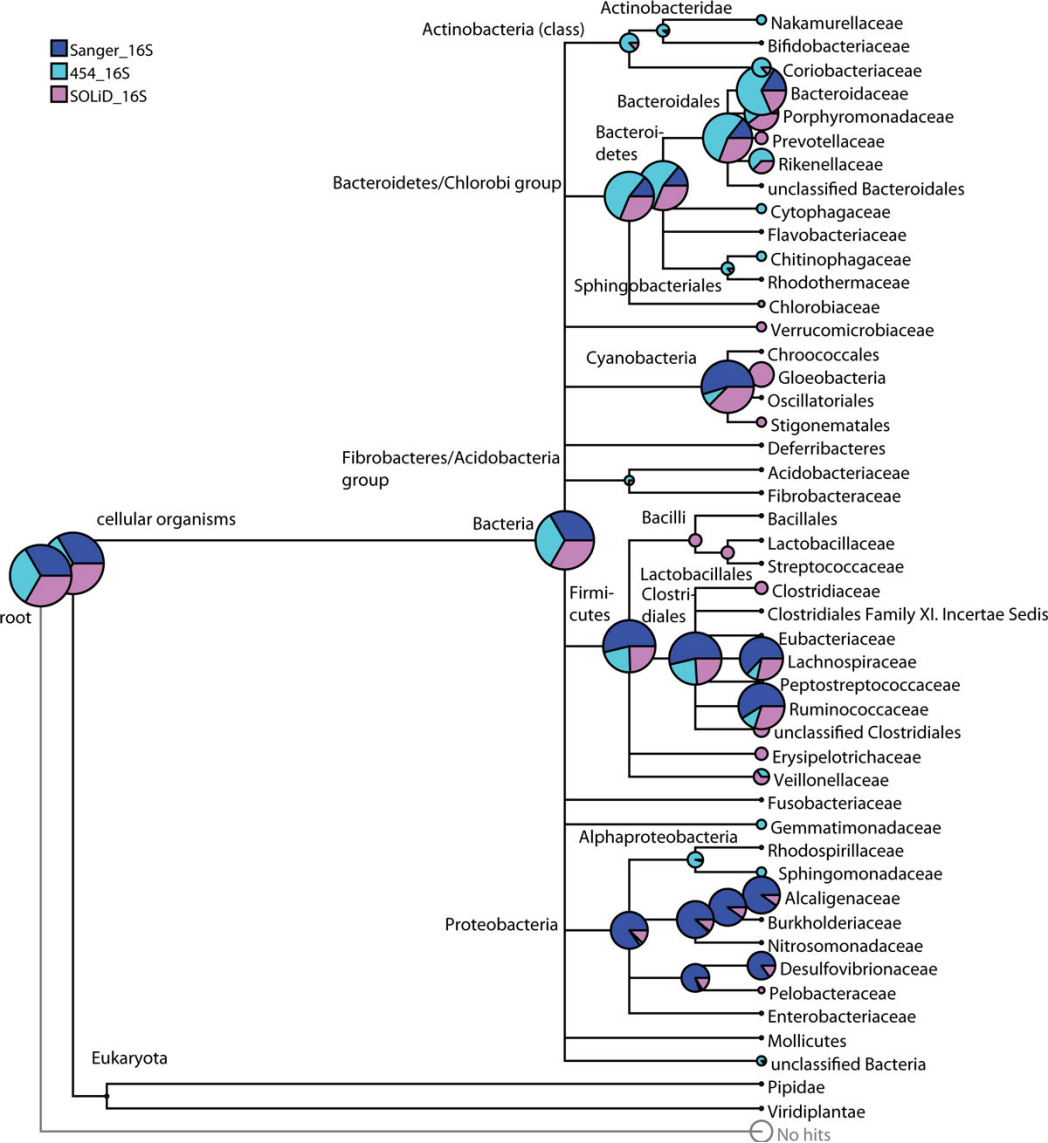
**Normalized comparison between 16S samples obtained using three technologies: 'Sanger', '16S-454' and '16S-SOLiD' datasets**. Normalized comparison result obtained using MEGAN for 'Sanger'-dataset (blue), '16S-454' dataset (cyan) and '16S-SOLiD' dataset (magenta) without considering 'No hits' node. The tree is collapsed at 'family' level of NCBI taxonomy. Circles are scaled logarithmically to indicate the number of summarized reads.

As SOLiD sequencing is substantially more cost-efficient than Sanger sequencing, it is possible to produce many more SOLiD reads at a very small fraction of the cost of a Sanger run. SOLiD sequencing produces very short sequences and many of them cannot be assigned, and these are shown as 'No hits' node in the above figures. Sanger sequencing does not have this limitation and 454 data are also less affected in this respect. Hence, we ignored the 'No hits' node in the comparison. Figure 3 depicts a normalized comparison tree view of the all the 16S samples obtained from three technologies at 'Family' level of the NCBI taxonomy. To facilitate visual comparison, nodes are scaled by 'summarized reads', that is, the number of reads assigned to or below a given node. It is clearly visible that we were able to find many phyla, such as *Actinobacteria*, and the domain of *Archaea *using SOLiD sequencing that were not detected by Sanger sequencing and appeared only with a few reads in the 454 dataset. Furthermore, important bacterial groups such as *Verrucomicrobia*, Lactobacilli, *Fusobacteria *and special members of the *Clostridiales *were not found by Sanger sequencing at all. In the 454 sample we detected *Verrucomicrobia*, but not the other two. We found comparable amounts between Sanger and 16S rRNA SOLiD sequencing for one the two major phyla of the intestinal microbiome, the *Baceriodetes *(Figure 3, Figure 1).

A detailed absolute comparison between 1242 16S-Sanger reads, 72571 reads of 16S-454 and the 300,000 reads from '16S SOLiD' dataset is depicted in Additional file 3. Here we can see that 300,000 reads of '16S-SOLiD' datasets already provides much resolution in the analysis when compared to 16S sequences from Sanger or 454 technologies. Furthermore, according to Sanger sequencing reads, assignments to phyla such as the *Proteobacteria *and the *Firmicutes *are dominant, possibly because of easier cloning and particular amplification procedures. This amplification process could be the cause for the differences seen when comparing the amounts of *Bacteroides*, *Gammaproteobacteria*, *Alphaproteobacteria *and Bacilli in 16S sequencing. It was already shown in Figure 2 that they are highly present in the shotgun dataset. Furthermore, the SOLiD datasets give information about the abundance of potentially pathogen microorganisms like *Camphylobacter*, *Listeria *and *Neisseria*. In the 'Sanger' dataset, these organisms were not detected due to their low abundance. The overrepresentation of the *Cyanobacteria *in the Sanger dataset was much less pronounced in the '16S-SOLiD' dataset. In the 'Sanger' dataset, the *Cyanobacteria *were the dominant group and had more reads than all other bacteria. In the '16S-SOLiD' dataset, they were still a group with a high abundance but the other bacterial groups were well represented, too. Low abundance of *Cyanobacteria *in the 'Shotgun-SOLiD' dataset could be explained by the missing amplification process in SOLiD technology. The advantage of SOLiD sequencing over Sanger sequencing is visible here. Due to the large number of reads, the overrepresentation of a bacterial group was less pronounced. Furthermore, the shotgun approach has the advantage of the avoiding amplification preferences for some bacterial groups. Figure 2 illustrates that the bacterial groups of *Actinobacteria*, *Bacteroidetes*, *Bacilli*, *Alpha- *and *Gammaproteobacteria *and *Clostridiaceae *are underrepresented when amplification processes were used.

Furthermore, paired reads using SOLiD technology achieved much more resolution than 454 single reads at a lower cost (see Additional file 4).

In total, these data suggest that SOLiD sequencing is a viable and cost efficient option for the analysis of the intestinal microbiome in spite of the short read length.

### Functional analyzes using SEED and KEGG

In this classification, genes are assigned to functional roles and different functional roles are grouped into subsystems. The SEED classification can be represented as a rooted tree in which internal nodes represent different subsystems and where leaves represent functional roles. MEGAN's functional analyzes using SEED classification is shown in Additional file 5.

For pathway analysis using KEGG, the program MEGAN matches each read to a KEGG orthology (KO) accession number, using the best hit to a reference sequence for which a KO accession number is known. The program reports the number of hits to each KEGG pathway. Additional file 6 depicts the result of such an analysis at the highest level of the KEGG hierarchy. To perform a functional analysis, MEGAN assigns each read to the functional role of the highest scoring gene in a BLAST or similar comparison against a protein database. To perform a KEGG analysis, then it attempts to match each read to a KEGG orthology (KO) accession number, using the best hit to a reference sequence for which a KO accession number is known. Thus from the functional analyses we can be informed about the possibility of metabolisms to be active. Thus this KEGG analysis is technically preliminary; therefore only a detailed examination of individual pathways will allow on to decide which pathways are actual active.

### Comparison with other approaches

To evaluate the performance of the MEGAN4 analysis based on a BLASTN comparison of the reads against the SILVA database, we ran the data through the RDP classifier [22](using 'Confidence threshold': 80%) (see Additional file 7). For RDP, we didn't specify minimum alignment length in order to allow all the assignments with previous threshold. The MEGAN analysis resulted in very similar annotation as with RDP. We also analyzed the data using MOTHUR software [23]. However, MOTHUR uses a simple best-hit assignment strategy that assigns all reads to the leaves of the NCBI taxonomy, regardless of the presence of other, equally similar reference sequences. Hence, a direct comparison against analyses performed using the LCA approach is hardly possible.

Beside these analyses an overall diversity was compared at genus level of the both 16S-SOLiD and 16S-454 data, using the Shannon-Weaver index and Simpson Reciprocal index, a measurement that combines diversity (the number of different nodes at a certain level) and evenness (the relative abundance of each node). Considering all the nodes at 'genus' level, we obtained for 16S SOLiD data Shannon and Simpson index values of 2.212 and 2.879, respectively. For 16S-454 data these two indices attain much lower values of 1.220 and 1.845, respectively.

## Discussion

The number of studies in which the composition of the intestinal microbiome is analyzed is growing rapidly. This is mainly due to continuous improvements to next generation sequencing technologies. The growth also reflects an increasing interest in understanding the function of the intestinal microbiome in health and disease [24,25]. Most previous studies on intestinal microbial community analysis were performed using 454 sequencing, a technique released early and resulting in relatively long read lengths. In 2010, Qin et al. [8] showed that metagenomic analysis of the intestinal microbiota can be performed using Illumina genome analyzer. Recently, Iverson et al. [26] demonstrated, that short-read mate-paired SOLiD sequencing is also capable of characterizing members of complex marine communities. In the present study, we show for the first time that the analysis of the human intestinal microbiome is also possible using the Applied Biosystems SOLiD sequencing platform.

To examine the eligibility of SOLiD sequencing for metagenomic approaches, we compared the most classical sequencing approach, 16S rRNA gene Sanger sequencing, and also the widely used 454 amplicon sequencing of the V2-region of the 16S rRNA gene with 16S rRNA gene sequencing and shotgun sequencing on the SOLiD platform. By focusing on the two most important phyla in the human gut, the *Firmicutes *and the *Bacteroidetes*, we found similar amounts of *Firmicutes *for Sanger and shotgun sequencing and also of *Bacteriodetes *for Sanger and 16S rRNA sequencing, respectively. The results for the 454 dataset lay between those for the other datasets with a slightly predominance of *Bacteroidetes *compared to *Firmicutes*. We suspect that the high amount of *Cyanobacteria *that was found in the 'Sanger' dataset is likely due to cloning or amplification artifacts. In the '16S-SOLiD' and '16S-454' dataset, the *Cyanobacteria *are reduced and in the 'Shotgun-SOLiD' dataset, they are present only at a marginal amount. However, it is unquestioned that *Cyanobacteria *are present in the analyzed fecal sample. These *Cyanobacteria *could represent descendants of nonphotosynthetic ancestral *Cyanobacteria *that have adapted to life in mammal intestines since previous studies also showed the presence of *Cyanobacteria *in the human and murine intestine, respectively [27,28]. Moreover, we found that most of the *Cyanobacteria *we found in the human fecal sample belong to *Gleobacter *a bacterial species that have no thylakoids (site of light-dependent reactions of photosynthesis) and lacks many genes of photosystem I and II [29]. However, the high amounts of *Cyanobacteria *found by Sanger sequencing might result from a particular technical feature, since the necessary cloning process could favor some 16S rRNA genes over others. In this case, it seems that *Cyanobacterial *16S rRNA genes are easier to clone and dominate over 16S rRNA genes from other species. This may also explain the over-representation of these organisms. Alternatively, these sequences might represent plant plastid sequences. However, by checking the reads assigned to the *Cyanobacteria *in detail, no hint to plant plastid sequences was found. Moreover, by inspection of the SEED and KEGG analysis no sequences assigned to photosynthesis were found.

One of the few relevant differences in results obtained by using various techniques is that the dominating phylum in the 16S rRNA SOLiD dataset was *Firmicutes*, while for shotgun sequencing *Bacteroidetes *were dominating. For this discrepancy, several explanations can be considered, which are not mutually exclusive. First, for the amplification of 16S rRNA genes universal bacterial primers were used, which are not able to catch all species while shotgun sequencing catches every single species present within the sample. This could explain the higher amount of hits for *Proteobacteria *and the presence of *Cyanobacteria *we described. Secondly, two different databases were used for bacterial analysis, the SILVA database for the 16S rRNA and the NCBI-NR database for the shotgun data. While the SILVA database is a 16S rRNA gene database, the NCBI-NR database also includes protein sequences for the metabolic information. The use of the NCBI-NR database explains the high number of "no hits" sequences in the shotgun dataset. Due to the short read lengths and the millions of entries, a lot of reads were not assignable with the used parameters. Previous studies revealed that there are high intra-individual differences regarding the dominating phylum, either *Bacteroides *or *Firmicutes *[6]. Therefore, the shotgun dataset, in which all species are detectable, might be more useful. This assumption is supported by the fact that *Actinobacteria*, *Lactobacillales*, *Clostridiaceae*, *Eubacteriaceae*, *Gammaproteobacteria*, families of the *Betaproteobacteria*, and *Archea *are even better detectable by shotgun sequencing instead of 16S sequencing. However, there are caveats to shotgun sequencing, too. Especially the amplification cycles that are used for SOLiD sequencing could cause GC-bias and thus *Firmicutes *with their known low GC-content could be underestimated. By taking a closer look at both next generation 16S rRNA gene datasets, it is obvious that in the 454 dataset the *Bacteroidetes *while in the 16S SOLiD dataset the *Firmicutes *were dominating. However, the absolute amounts of *Firmicutes *in these both datasets are nearly the same. The main difference we observed is the various amounts of *Cyanobacteria *detected depending on the methods used. We found many reads assigned to the *Cyanobacteria *in the 16S SOLiD dataset but much less in the 454 dataset. This difference could be caused by amplification and sequencing of only the V2-region of the 16S rRNA gene in the 454 approach and the whole 16S rRNA gene in the SOLiD 16S approach.

Overall, we show that the different sequencing techniques yield similar albeit not identical results, and that intestinal microbiome analyzed in the present study is similar composed as other microbiomes analyzed in previous studies [6,28,30].

Regardless of the many "no hits" sequences found by SOLiD shotgun sequencing, a major advantage of this approach is that it delivers metabolic information not available with 16S rRNA sequencing. Indeed, the metabolic dataset provides information about all coded proteins relevant for the microbial metabolism. In this study, two different functional analyzes were used: MEGAN's KEGG-based pathway analysis and the MEGAN's SEED-based functional analysis. The high amount of functional genes for carbohydrate and energy metabolism that is shown by both functional analyzes in this sample is not surprising. Our data confirm previous findings showing that in obesity-associated intestinal microbiomes the number of metabolizing enzymes and the capacity for energy harvest is enhanced relative to the microbiomes of lean people [31]. Furthermore, typical microbial metabolic features such as the biosynthesis of secondary metabolites, transport, xenobiotics degradation, and glycan biosynthesis are detected. This metabolic information will be particularly helpful for the characterization of functional differences in intestinal microbiomes from lean and obese individuals. The detected KEGG human diseases pathways are low-numbered in comparison to the detected microbial pathways. In all likelihood, the sequences come from human DNA that was isolated together with the microbial DNA. A part of the "*eukaryota*" hits in the shotgun datasets was human, too. Using the whole extracted DNA for shotgun sequencing carryover of host DNA is nearly unavoidable.

Following the manufactures protocols for high-throughput 96-well Sanger sequencing using ABI BigDyeTerminator and 3730xl capillary electrophoresis in comparison to massive parallel SOLiD Mate-Paired-Sequencing on one 1/8 part of one SOLiD slide, we were able to reduce the cost of the metagenomic analysis for the portion of informative reads by the factor 2,000. This saving can be utilized to improve coverage and deepness of the analysis on one hand, and to reduce overall costs of any metagenomic analysis on the other hand. Taken into account that the price per kb for Sanger-derived sequences reached a plateau for several years, and massive parallel SOLiD sequencing costs will drop even more in the near future, it is well foreseeable that a huge number of metagenome sequencing projects will become a realistic scenario also in a diagnostic setting using next generation sequencing platforms. Most importantly, massive parallel multiplexed sequencing and longer mate-pair libraries will achieve more flexibility, which will give the scientist the opportunity to balance the need of deepness for the specific project.

## Conclusions

Here, we investigated the performance of SOLiD sequencing by comparing taxonomic profiles of SOLiD mate-pair sequencing reads with Sanger paired reads and 454 single reads obtained from the bacterial 16S rRNA gene, which was amplified from microbial DNA extracted from a human fecal sample. Moreover, the complete genomic microbial DNA was shotgun sequenced using SOLiD sequencing. The microbiota composition of 16S rRNA gene sequences obtained using Sanger, 454 and SOLiD sequencing provided results comparable to the result based on shotgun sequencing. Furthermore, SOLiD sequences provided more resolution down to the species level. Thus, SOLiD mate-pair sequencing seems to be a viable and cost-efficient option for analyzing a complex environment such as the human intestinal microbiome.

## Competing interests

The authors declare that they have no competing interests.

## Authors' contributions

KFF carried out the isolation of microbial DNA and amplification of 16S RNA, SB and TS carried out the Sanger and SOLiD Long-Mate-Paired sequencing, SM was responsible for data processing. KFF, SM performed analysis and interpretation of data and drafted the manuscript. ADM participated in data analysis and helped to draft the manuscript. DHH and SCB participated in the design and coordination and revised the manuscript for important intellectual content. All authors read and approved the final manuscript.

## Supplementary Material

Additional file 1 **Normalized comparison tree view of four methods described in this paper**. Click here for file

Additional file 2 **Absolute comparison tree view of Shotgun-SOLiD datasets considering long (40+ bp) and small mates**. Click here for file

Additional file 3 **Absolute comparison tree view of 'Sanger'-dataset, '454'- dataset and a subset of the 'Shotgun-SOLiD'-dataset**. Click here for file

Additional file 4 **Detailed normalized comparison between 16S samples obtained using 454 and SOLiD sequencing**. Click here for file

Additional file 5 **SEED-based functional analysis of 'Shotgun-SOLiD' dataset**. Click here for file

Additional file 6 **KEGG-based pathway analysis of 'Shotgun-SOLiD' dataset**. Click here for file

Additional file 7 **Comparison of three different 16S analyses**. Click here for file

## Declarations

Publication of this article was funded by University of Hohenheim.

This article has been published as part of *BMC Genomics *Volume 14 Supplement 5, 2013: Twelfth International Conference on Bioinformatics (InCoB2013): Computational biology. The full contents of the supplement are available online at http://www.biomedcentral.com/bmcgenomics/supplements/14/S5.
