# Supplementary figures and images for: Analysis of the intestinal microbiota using SOLiD 16S rRNA gene sequencing and SOLiD shotgun sequencing
Source: BMC Genomics. 2013 Oct 16;14(Suppl 5):S16. doi: 10.1186/1471-2164-14-S5-S16 (PMC3852202; doi:10.1186/1471-2164-14-S5-S16)

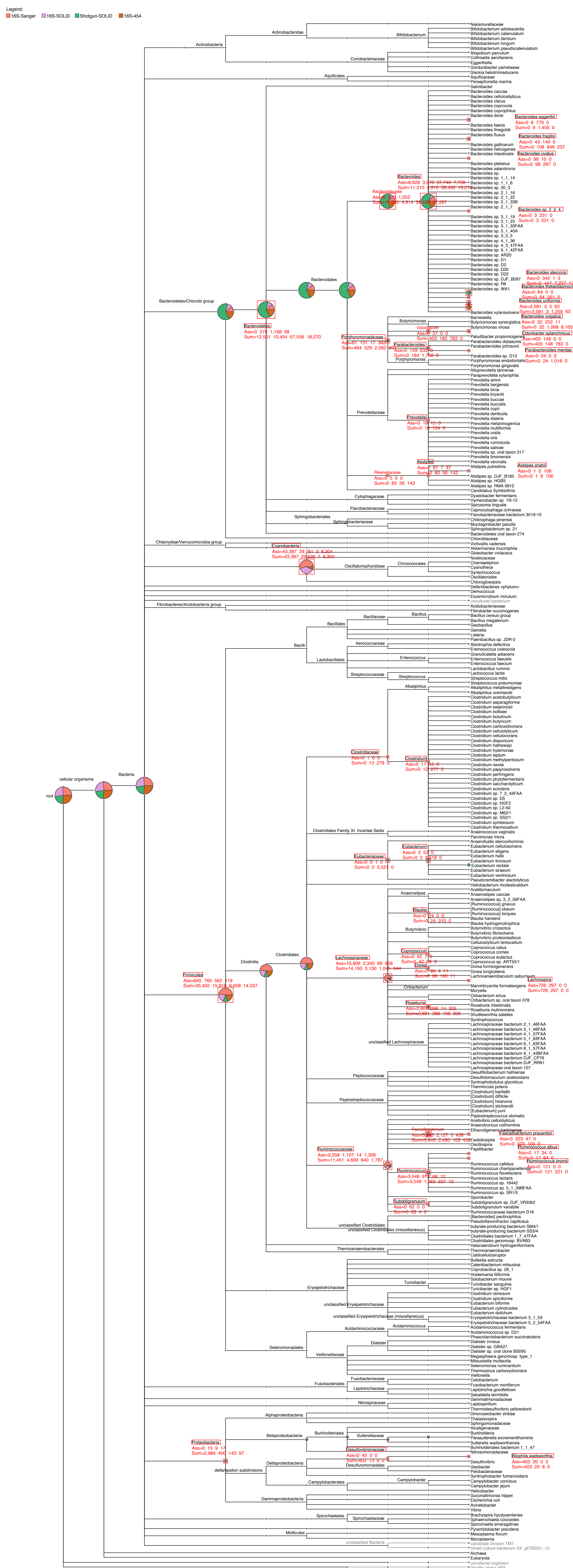

Supplement: Additional file 1 — Normalized comparison tree view of four methods described in this paper. [file 1471-2164-14-S5-S16-S1.pdf]
