# Supplementary material for: Analysis of the intestinal microbiota using SOLiD 16S rRNA gene sequencing and SOLiD shotgun sequencing
Source: BMC Genomics. 2013 Oct 16;14(Suppl 5):S16. doi: 10.1186/1471-2164-14-S5-S16 (PMC3852202; doi:10.1186/1471-2164-14-S5-S16)
Supplement: Additional file 2 — Absolute comparison tree view of Shotgun-SOLiD datasets considering long (40+ bp) and small mates. [file 1471-2164-14-S5-S16-S2.pdf]

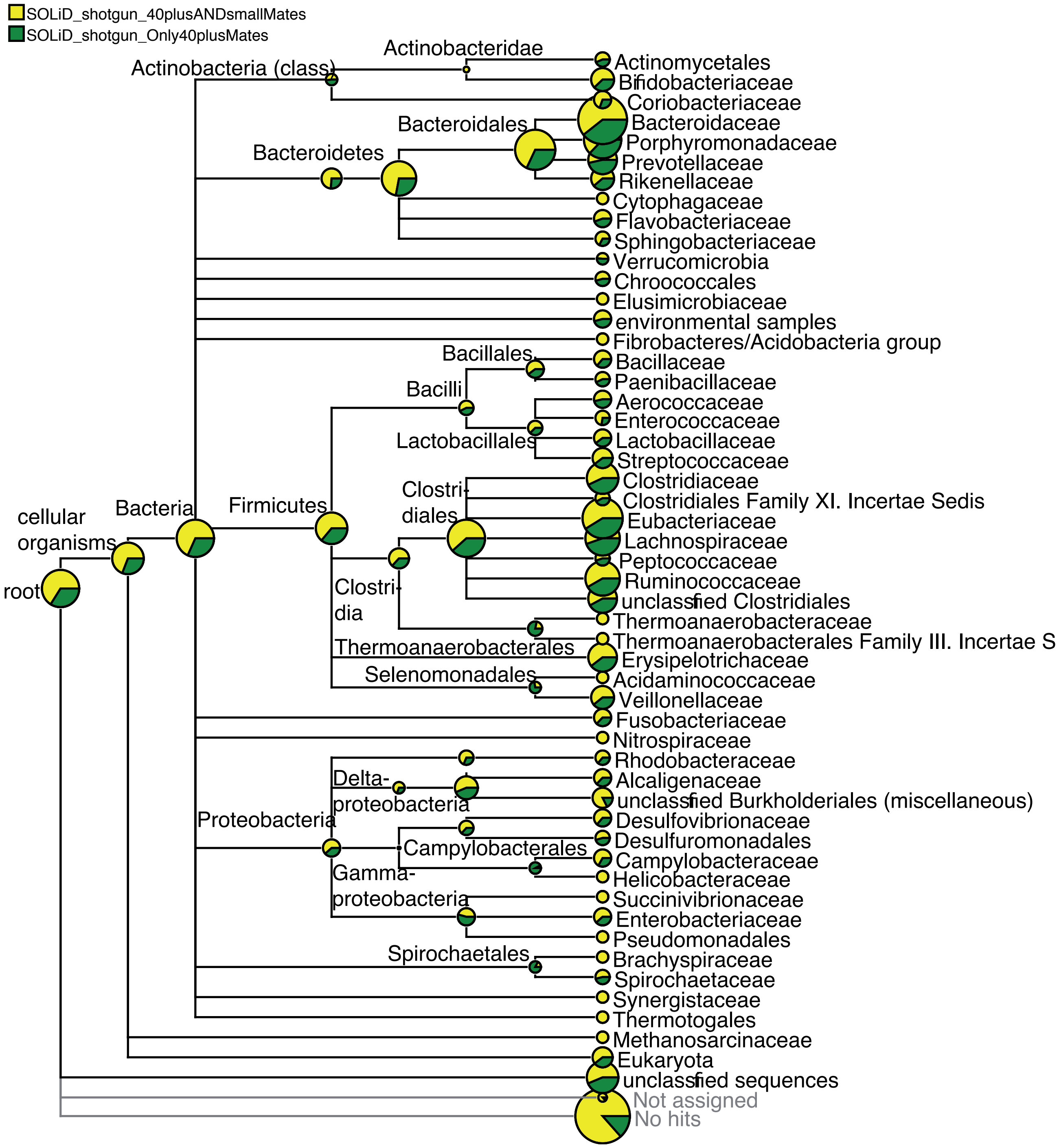

Additional Figure 2: Absolute comparison tree view of Shotgun-SOLiD datasets considering long (40+ bp) and small mates. Absolute comparison tree view using MEGAN 4 of 'Shotgun-SOLiD' datasets when considering all the mates for which both reads have a length of at least 40 bp (green) against all the mates for which at least one of the read is 40 bp or more (yellow).
