# Supplementary material for: Analysis of the intestinal microbiota using SOLiD 16S rRNA gene sequencing and SOLiD shotgun sequencing
Source: BMC Genomics. 2013 Oct 16;14(Suppl 5):S16. doi: 10.1186/1471-2164-14-S5-S16 (PMC3852202; doi:10.1186/1471-2164-14-S5-S16)
Supplement: Additional file 4 — Detailed normalized comparison between 16S samples obtained using 454 and SOLiD sequencing. [file 1471-2164-14-S5-S16-S4.pdf]

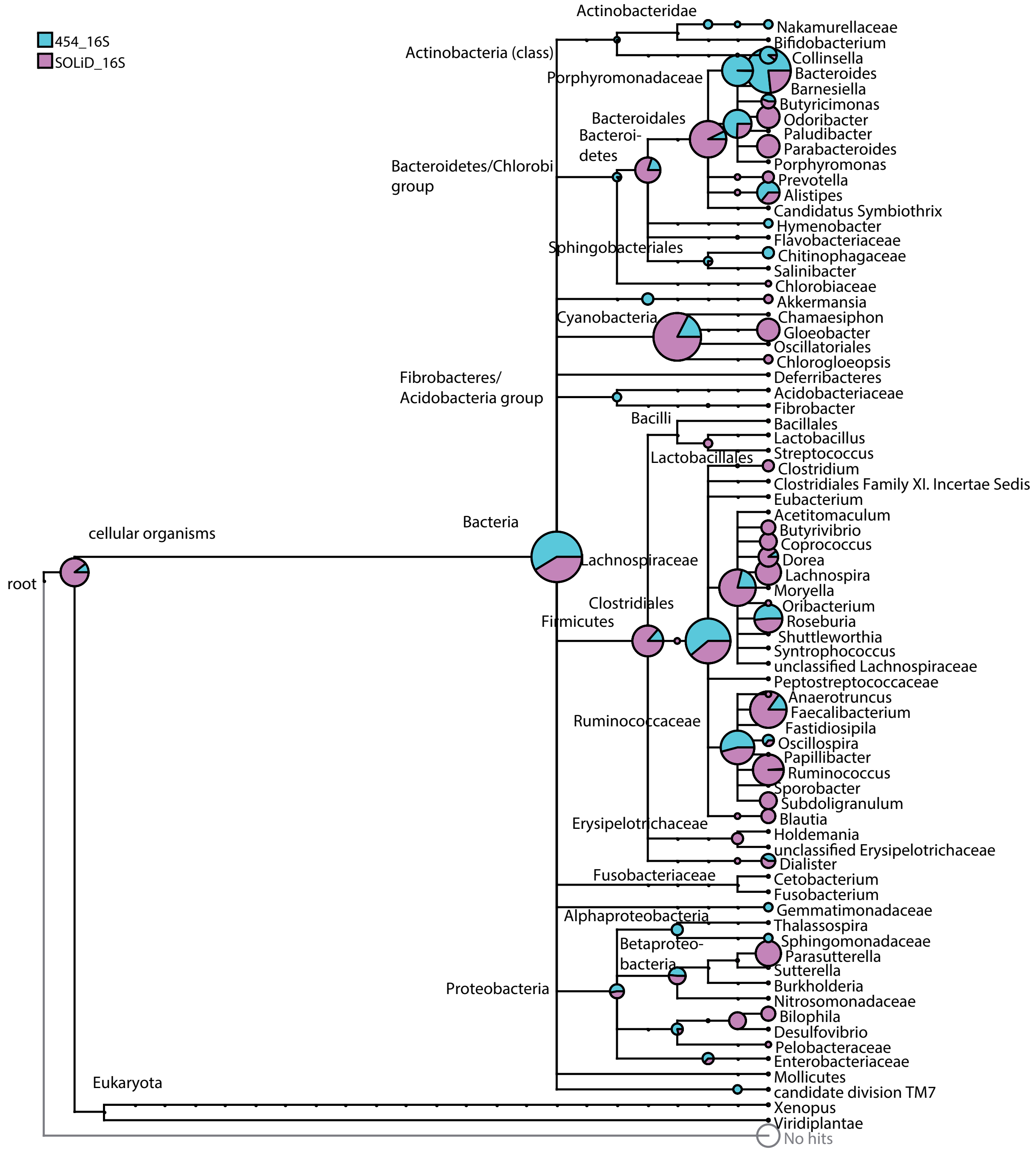


**Additional Figure 4: Detailed normalized comparison between 16S samples obtained using 454 and SOLiD sequencing.** Detailed normalized comparison tree obtained using MEGAN for '16S-454' dataset (cyan) and '16S-SOLiD' dataset (magenta) without considering the 'No hits' node. The tree is collapsed at 'genus' level of NCBI taxonomy. Circles are scaled logarithmically to indicate the number of summarized reads.
