# Supplementary material for: Analysis of the intestinal microbiota using SOLiD 16S rRNA gene sequencing and SOLiD shotgun sequencing
Source: BMC Genomics. 2013 Oct 16;14(Suppl 5):S16. doi: 10.1186/1471-2164-14-S5-S16 (PMC3852202; doi:10.1186/1471-2164-14-S5-S16)
Supplement: Additional file 5 — SEED-based functional analysis of 'Shotgun-SOLiD' dataset. [file 1471-2164-14-S5-S16-S5.pdf]

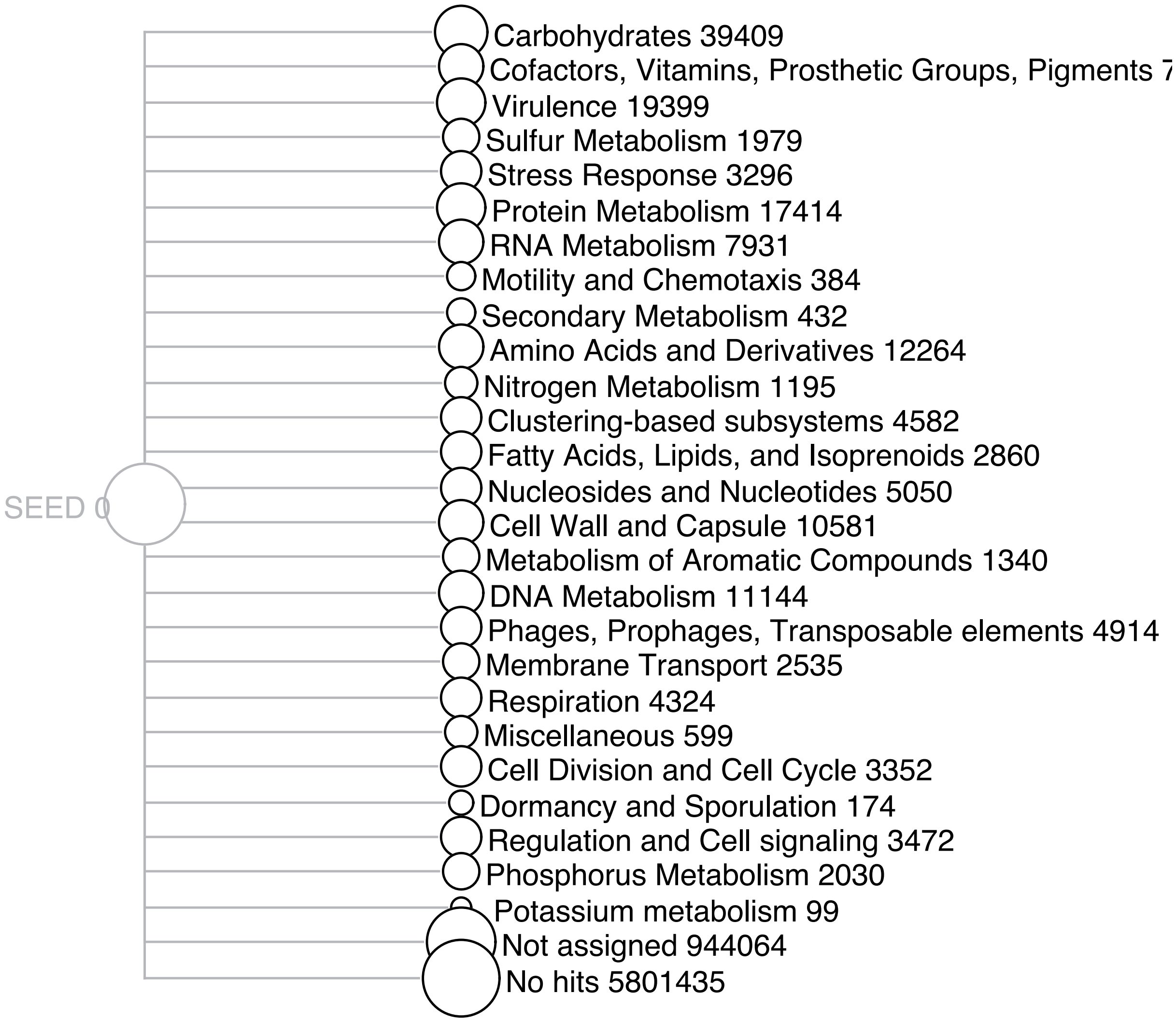

Additional Figure 5: SEED-based functional analysis of 'Shotgun-SOLiD' dataset. SEED-based functional analysis of 'Shotgun-SOLiD' dataset computed using MEGAN. Numbers in the tree indicate the number of assigned reads to each SEED subsystems. Different functional roles are grouped into subsystems. One read can be assigned to more than one functional role. Circles are scaled logarithmically to indicate the number of assigned of reads.
