# Supplementary material for: Analysis of the intestinal microbiota using SOLiD 16S rRNA gene sequencing and SOLiD shotgun sequencing
Source: BMC Genomics. 2013 Oct 16;14(Suppl 5):S16. doi: 10.1186/1471-2164-14-S5-S16 (PMC3852202; doi:10.1186/1471-2164-14-S5-S16)
Supplement: Additional file 6 — KEGG-based pathway analysis of 'Shotgun-SOLiD' dataset. [file 1471-2164-14-S5-S16-S6.pdf]

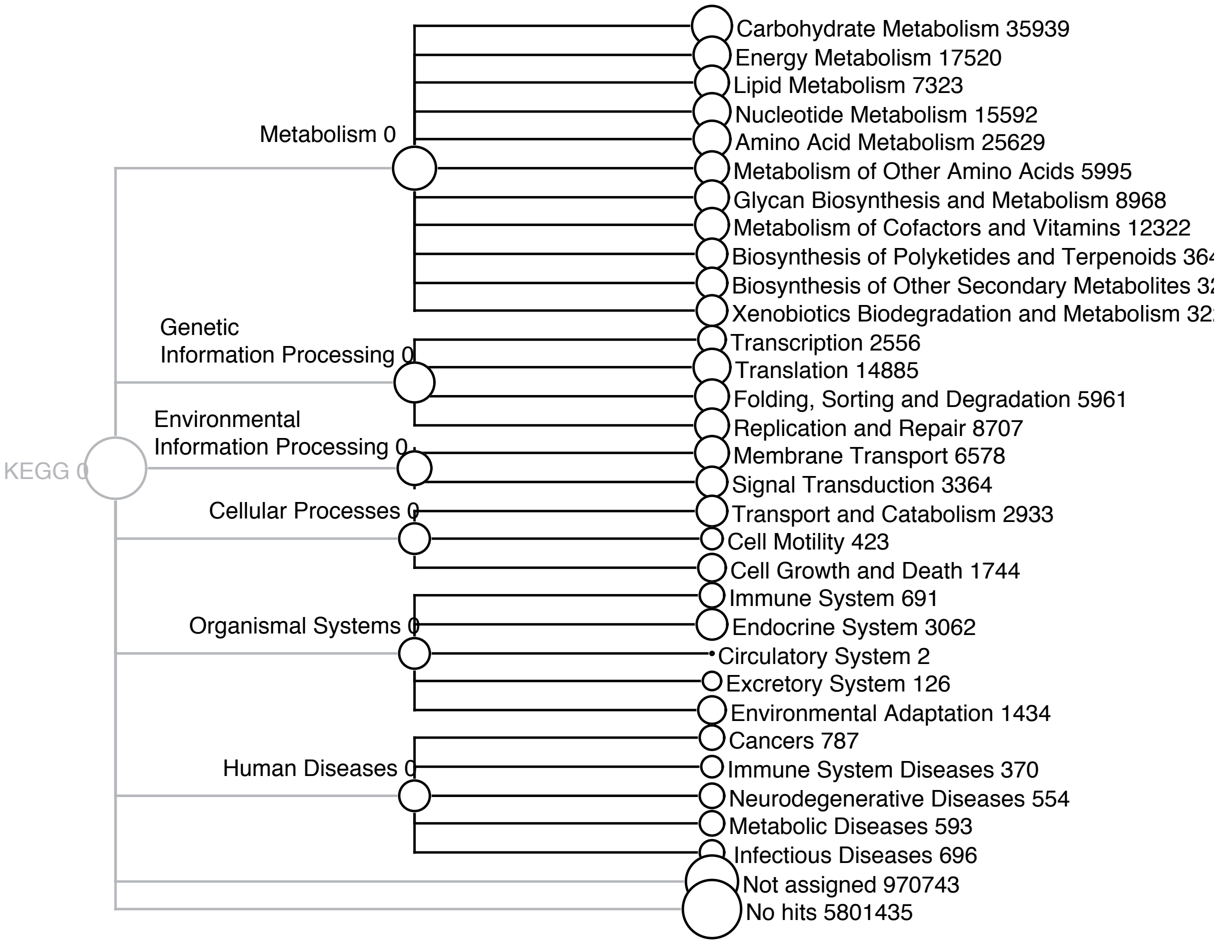

Additional Figure 6: KEGG-based pathway analysis of 'Shotgun-SOLiD' dataset. KEGG-based pathway analysis of 'Shotgun-SOLiD' dataset computed using MEGAN. Numbers in the tree indicate the number of assigned reads to each KEGG pathways. Circles are scaled logarithmically to indicate the number of assigned of reads.
