# Supplementary material for: Analysis of the intestinal microbiota using SOLiD 16S rRNA gene sequencing and SOLiD shotgun sequencing
Source: BMC Genomics. 2013 Oct 16;14(Suppl 5):S16. doi: 10.1186/1471-2164-14-S5-S16 (PMC3852202; doi:10.1186/1471-2164-14-S5-S16)
Supplement: Additional file 7 — Comparison of three different 16S analyses. [file 1471-2164-14-S5-S16-S7.pdf]

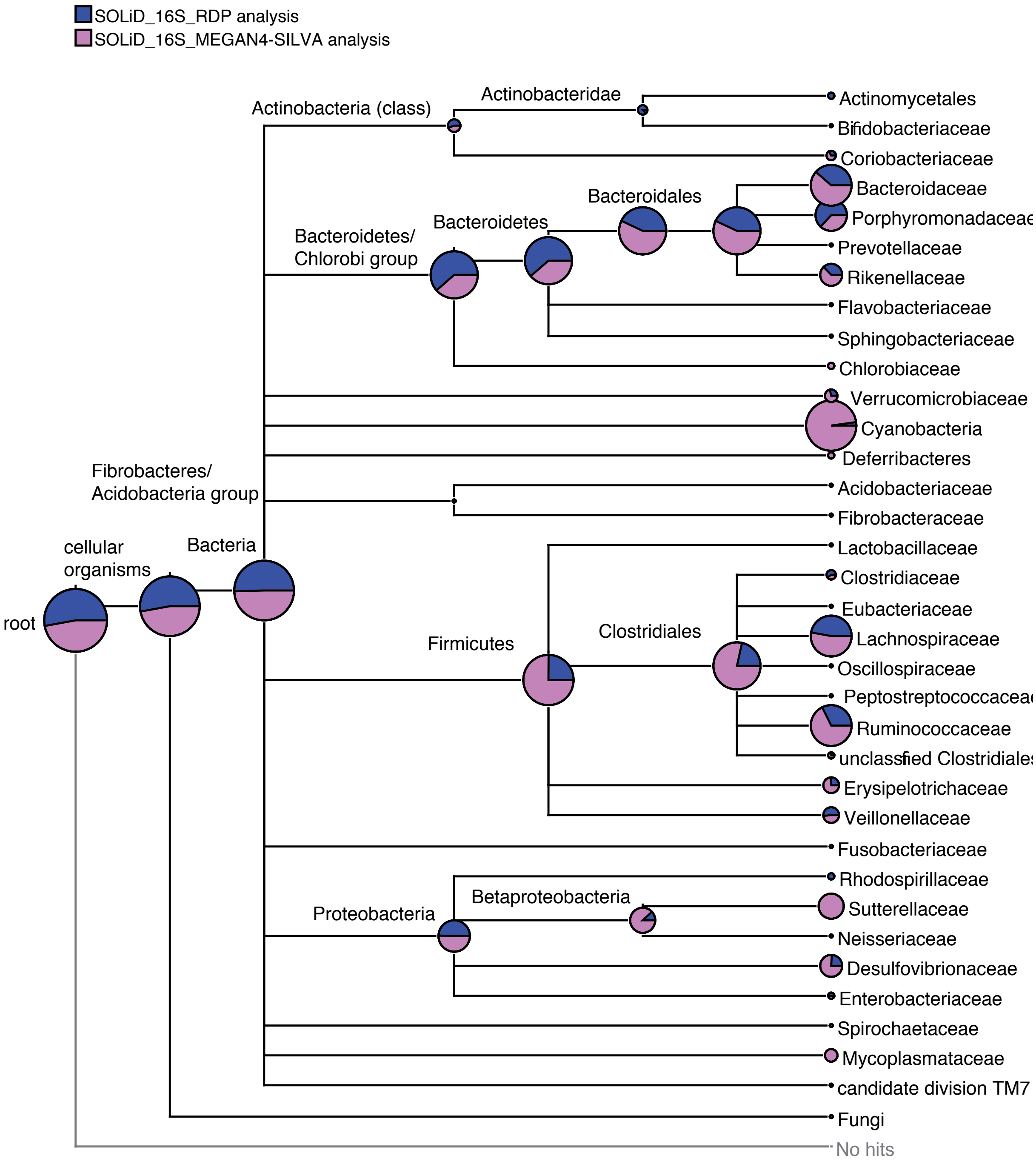

Additional Figure 7: Comparison of three different 16S analyses. Comparison of a MEGAN4 analysis based on a BLASTN comparison of the reads against the SILVA database (magenta) with a RDP analysis (blue).
