# Supplementary material for: Analysis of the intestinal microbiota using SOLiD 16S rRNA gene sequencing and SOLiD shotgun sequencing
Source: BMC Genomics. 2013 Oct 16;14(Suppl 5):S16. doi: 10.1186/1471-2164-14-S5-S16 (PMC3852202; doi:10.1186/1471-2164-14-S5-S16)
Supplement: Additional file 3 — Absolute comparison tree view of 'Sanger'-dataset, '454'- dataset and a subset of the 'Shotgun-SOLiD'-dataset. [file 1471-2164-14-S5-S16-S3.pdf]

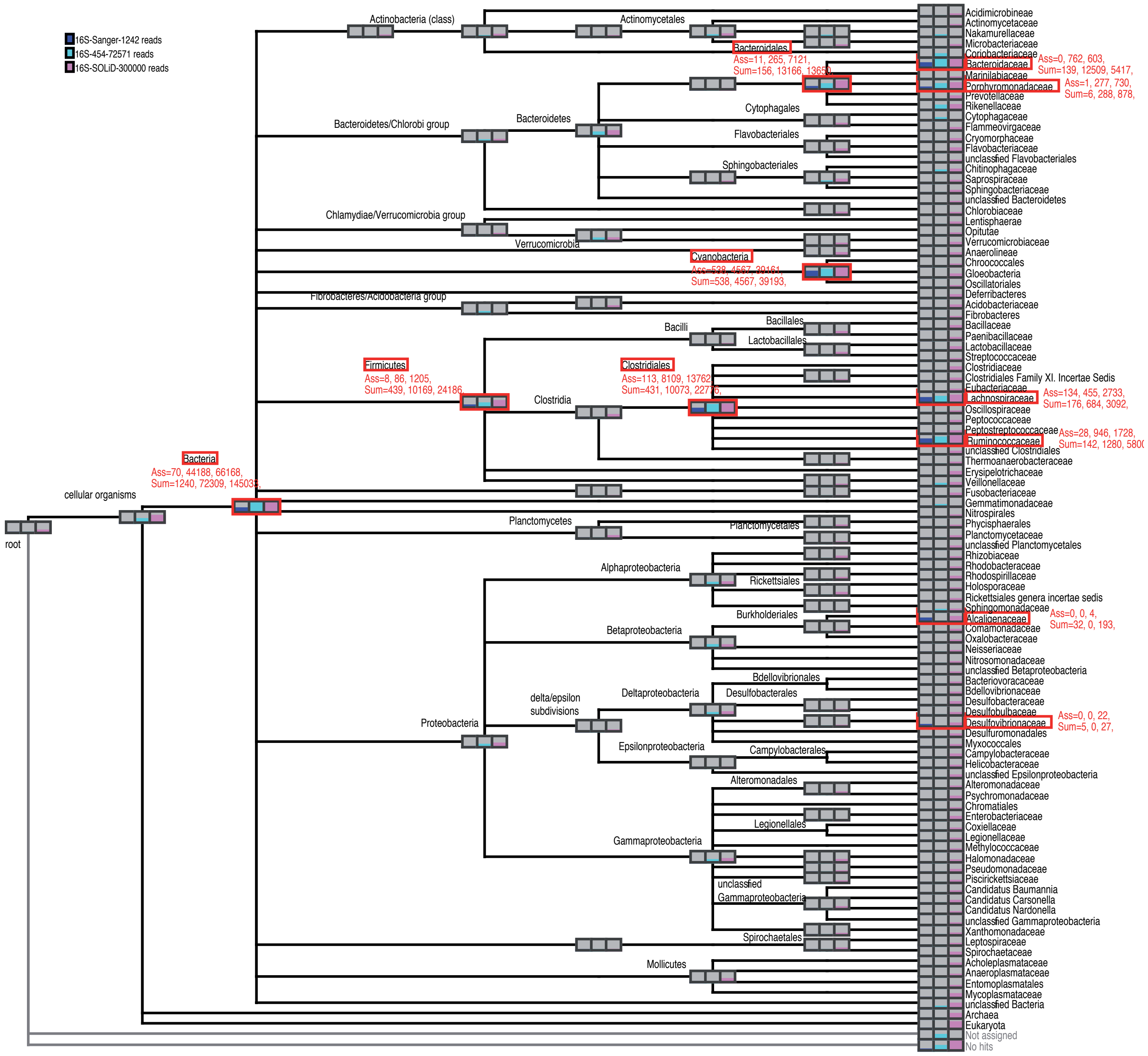

Additional Figure 3: Absolute comparison tree view of 'Sanger'-dataset, '454'- dataset and a subset of the 'Shotgun-SOLiD'-dataset. Absolute comparison tree view of 'Sanger'-dataset (blue, 1,242 sequences) and '454'-dataset (cyan, 72,571 sequences) with a subset (300,000 sequences) of the 'Shotgun-SOLiD' dataset (magenta), using MEGAN 4. The tree is collapsed at 'family' level of NCBI taxonomy.
